# Partnering With African American Faith-Based Communities to Support Dementia Caregivers

**DOI:** 10.1093/geroni/igab046.342

**Published:** 2021-12-17

**Authors:** Monica Long, Shellie Williams, Katherine Thompson, Jason Molony, Jeff Graupner

**Affiliations:** 1 University of Chicago, Chicago, Illinois, United States; 2 University of Chicago Medicine, Chicago, Illinois, United States; 3 The University of Chicago Medicine, Chicago, Illinois, United States

## Abstract

African Americans (AA) are twice as likely to develop Alzheimer’s Disease as Caucasians. Historically, houses of faith have been a center of the AA community and a trusted source of information and support. Based on these facts, as well as community needs, the SHARE Network (a Geriatrics Workforce Enhancement Program on the South Side of Chicago) in partnership with faith-based community leaders, created an opportunity for community members to train to become resource experts on Alzheimer’s Disease & Related Dementias (ADRD) and create sustainable caregiver support groups (CSGs). The resulting initiative, Dementia Resource Champions, is a train-the-trainer style health education initiative piloted in 2018, and subsequently expanded and modified due to COVID. Participants receive instruction on stress reduction, ADRD, and community resources. They discuss how to structure CSGs to meet community needs. Results of this initiative include development of five brand-new CSGs with faith communities on Chicago’s South Side.

